# Current insights into PHF14: molecular features and functional roles

**DOI:** 10.3389/fmolb.2026.1858432

**Published:** 2026-08-21

**Authors:** Vaishnavi Gopalakrishnan, Amal Fahma, Rajesh Raju, Sowmya Soman

**Affiliations:** Centre for Integrative Omics Data Science (CIODS), Yenepoya (Deemed to be University), Mangalore, Karnataka, India

**Keywords:** cancer, epigenetic regulation, histone reader, molecular interactions, PHF14

## Abstract

Plant Homeodomain Finger Protein 14 (PHF14) is a known epigenetic regulator involved in chromatin-mediated gene regulation. PHF14 belongs to the plant homeodomain (PHD) family, and possesses four PHD zinc-finger domains. The N-terminal PHD1 and PHD2 domain of PHF14 mediate histone recognition through specific PHD1–ZnK–PHD2 (PZP) cassettes and thereby contribute to the regulation of transcriptional activation and gene silencing. PHF14 plays a role in regulating essential cellular processes such as the cell cycle, mesenchymal proliferation, DNA damage response, and B-cell proliferation. Genetic variants of PHF14 have been identified across multiple clinical conditions, underscoring its physiological and pathologic relevance. Over the past 5 years, PHF14 has garnered substantial attention for its dysregulation or sequence variation associated with various cancers. PHF14-mediated interactions are implicated in neurodevelopmental disorders and also contribute to lung and renal fibrosis, as well as Dandy–Walker syndrome, underscoring its role in diverse pathological conditions. This review summarizes current knowledge on PHF14 structure, interaction partners, molecular functions, associated signaling pathways, and roles in diseases.

## 1 Introduction

The Plant Homeodomain (PHD) family comprises around 100 proteins with histone reader function and is involved in regulating diverse processes through chromatin regulation (Jain et al., 2020). Specifically, they harbour PHD zinc finger domain (s) that recognize the N-terminal tail of histone H3 with methylated or unmethylated lysine 4 (H3K4), trimethylated lysine 9 (H3K9me3), and acetylated lysine 14 (H3K14) forms (Musselman and Kutateladze, 2011; Jain et al., 2020). These proteins play a significant role in mediating molecular interactions in gene transcription. PHD finger protein 14 (PHF14), one of the members of this family, is a histone-binding protein that reads unmodified histone 3, influences the cell cycle, and chromatin-mediated transcriptional regulation (Park et al., 2019; Zhao et al., 2020; Zheng et al., 2021). PHF14 has recently been shown to be overexpressed in cancers and implicated in the pathogenesis of cancer. Apart from cancer, recent research highlights the importance of PHF14-mediated interactions in neurodevelopmental disorders (Dominguez et al., 2024). Previous studies in mice have shown that Phf14 is indispensable for postnatal development and survival, as its loss leads to lethality. Histopathological analysis of Phf14 knockout mice revealed severe abnormalities in the cell and tissue architecture of multiple organs, particularly lungs. Depletion of Phf14 in mice resulted in poorly developed alveoli, impaired lung function, and neonatal death (Huang et al., 2013). Considering its importance in epigenetic regulation during embryonic development, the understanding of PHF14 becomes essential. This review discusses the structural architecture of PHF14 and explores its molecular interaction with other chromatin-associated complexes. It also outlines the mechanisms involved in the regulation of PHF14 and summarises its molecular functions. The present review highlights the major signaling pathways associated with PHF14 in various biological contexts. Furthermore, we discuss its pathological implications, including reported genetic variants, its role in cancer, fibrosis, and immune regulation.

## 2 Overview of PHF14

### 2.1 Structure

PHF14 was initially identified as KIAA0783 in a large-scale cDNA sequencing project, which focused on characterizing novel human genes that encode large proteins from brain cDNA libraries (Nagase et al., 1998). PHF14 is conserved from *Caenorhabditis elegans* to *Homo sapiens*, and its gene is located on chromosome 7p21.3 in humans (Huang et al., 2013). It has 948 amino acids with a molecular mass of approximately 106.99 kDa (Huang et al., 2013). PHD fingers are the characteristic feature of the PHD family. Structurally conserved PHD zinc fingers are zinc-coordinating domains found in chromatin-associated proteins that mediate molecular interactions in gene transcription (Sanchez and Zhou, 2011). The tryptophan residue in the PHD domain that recognizes H3K4 trimethylation is conserved (Jain et al., 2020).

**FIGURE 1:**
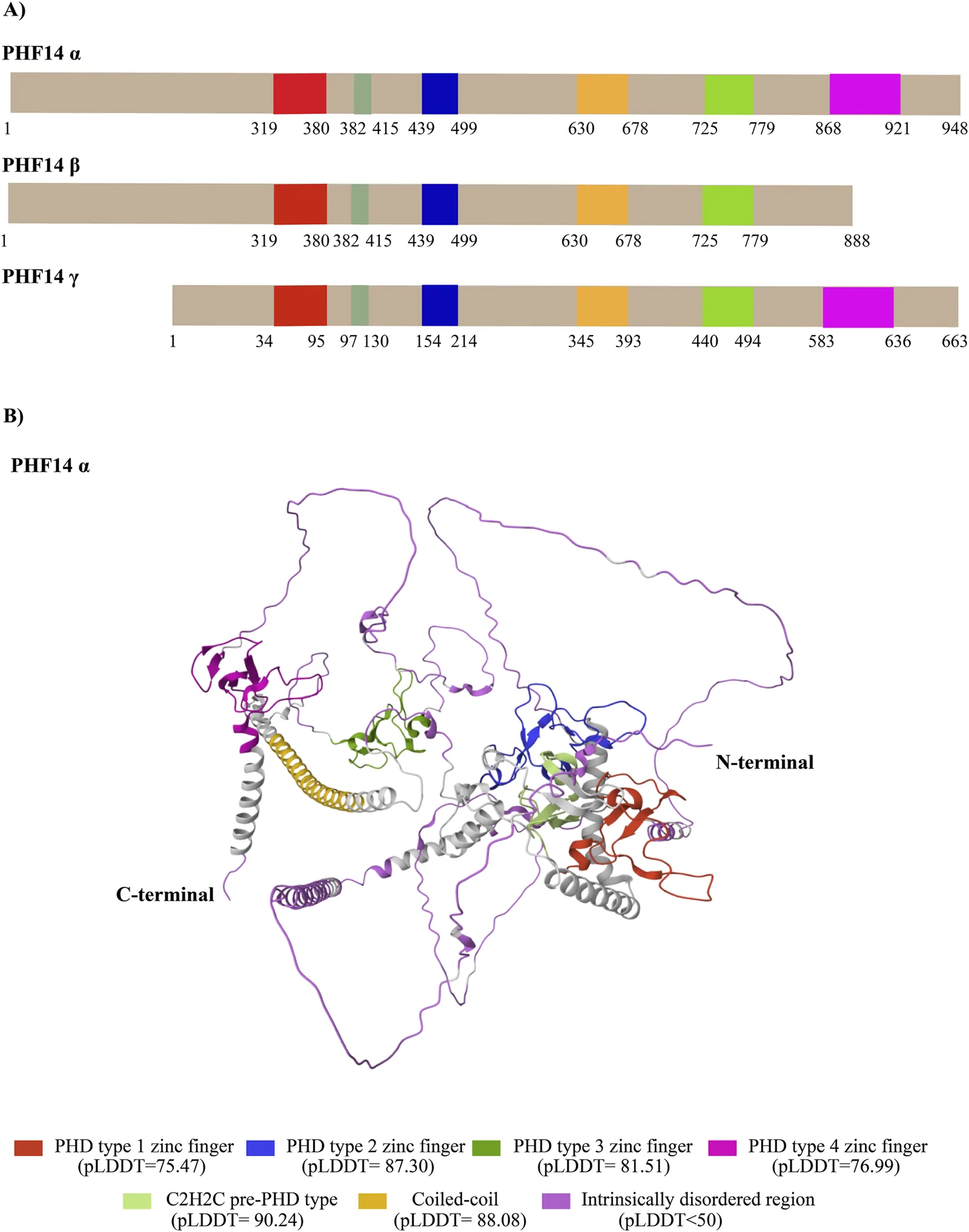
Protein structure of PHF14. **(A)** It represents the structural arrangement of PHD-type zinc finger, coiled coil region, and C2HC pre-PHD type in the three isoforms of PHF14 (α, β, γ). **(B)** Three-dimensional structure of PHF14 predicted by alphafold, highlighting PHD-type zinc finger. Domain annotations were retrieved from UniProt.

According to UniProt (UniProt Consortium, 2025), the structure of PHF14 consists of four PHD zinc fingers, one variant C2HC pre-PHD-type, and a coiled coil region (630–678). The gene *PHF14* produces three isoforms, namely, PHF14α (1–948), PHF14β (1–888), and PHF14*γ* (1–663) (Figure 1A). PHF14β lacks 60 amino acids at its C-terminal, whereas PHF14-gamma lacks 285 amino acids at its N-terminal. PHF14α is the longest isoform containing all four PHD fingers (Figure 1B). It is ubiquitously expressed, with high levels in the lungs, testis, spleen, and low levels in the heart, kidney, intestine, and muscle. PHF14α is dominantly expressed over PHF14β. PHF14α is found in the nucleus and binds chromatin during cell division, whereas PHF14β is present in the cytoplasm (Huang et al., 2013). PHFα was predicted to have 4 nuclear localization signals (NLS) and PHFβ to have 3 NLS. The isoform-specific functions of PHF14 remain largely unexplored, representing an important knowledge gap. The coiled-coil region (627–678) of PHF14 functions as a leucine zipper to interact with chromatin-associated proteins, such as High mobility group protein 20A (HMG20A) (Gómez-Marín et al., 2022). The first two PHD fingers of PHF14, such as PHD1 and PHD2, are closely connected by a single zinc knuckle, termed the PZP (PHD-zinc-knuckle-PHD) domain, which has been observed in protein families (Protein AF-10/17 (AF10/17), Lysine Demethylase 4 (KDM4), Bromodomain and PHD Finger-containing Protein (BRPF), and Protein Jade-1 (JADE)) of the human genome (Chen et al., 2015; Klein et al., 2020; Gaurav et al., 2024). The C-terminal region of PHF14 contains PHD3 and PHD4 fingers, which lack key residues for histone binding, and their exact function remains unclear (Zheng et al., 2021). The full-length crystal structure of PHF14 has not yet been determined; however, the structure of its PZP domain has been resolved in zebrafish (*Danio rerio*) (Zheng et al., 2021). This PZP domain mediates a bipartite binding mechanism and preferentially recognizes the unmodified (“ground-state”) histone H3. Structural analyses revealed that bipartite recognition of PHF14 mediates its interaction with two distinct regions of histone H3 N-terminal (1–15) and H3-middle (14–34) of H3(1–34) (Zheng et al., 2021). This interaction between PHF14 and Histone 3 depends on post-translational modifications (PTM) of the histone 3 tail. PTMs of residues R2, T3, K4, R8, and K23 interfere with PHF14 PZP binding, indicating that PHF14 specifically recognizes and prefers the unmodified, or “ground-state,” form of H3 at these positions. In contrast, this binding was not sensitive to K9 and K27 modifications. These modifications could act as a switch that regulates PHF14 binding with histone 3, and active epigenetic marks may negatively regulate its function (Zheng et al., 2021). This PZP-mediated bipartite recognition and preference for unmodified (“ground-state”) histone H3 sets it apart from other PHF proteins.

### 2.2 Molecular interactions of PHF14

**FIGURE 2:**
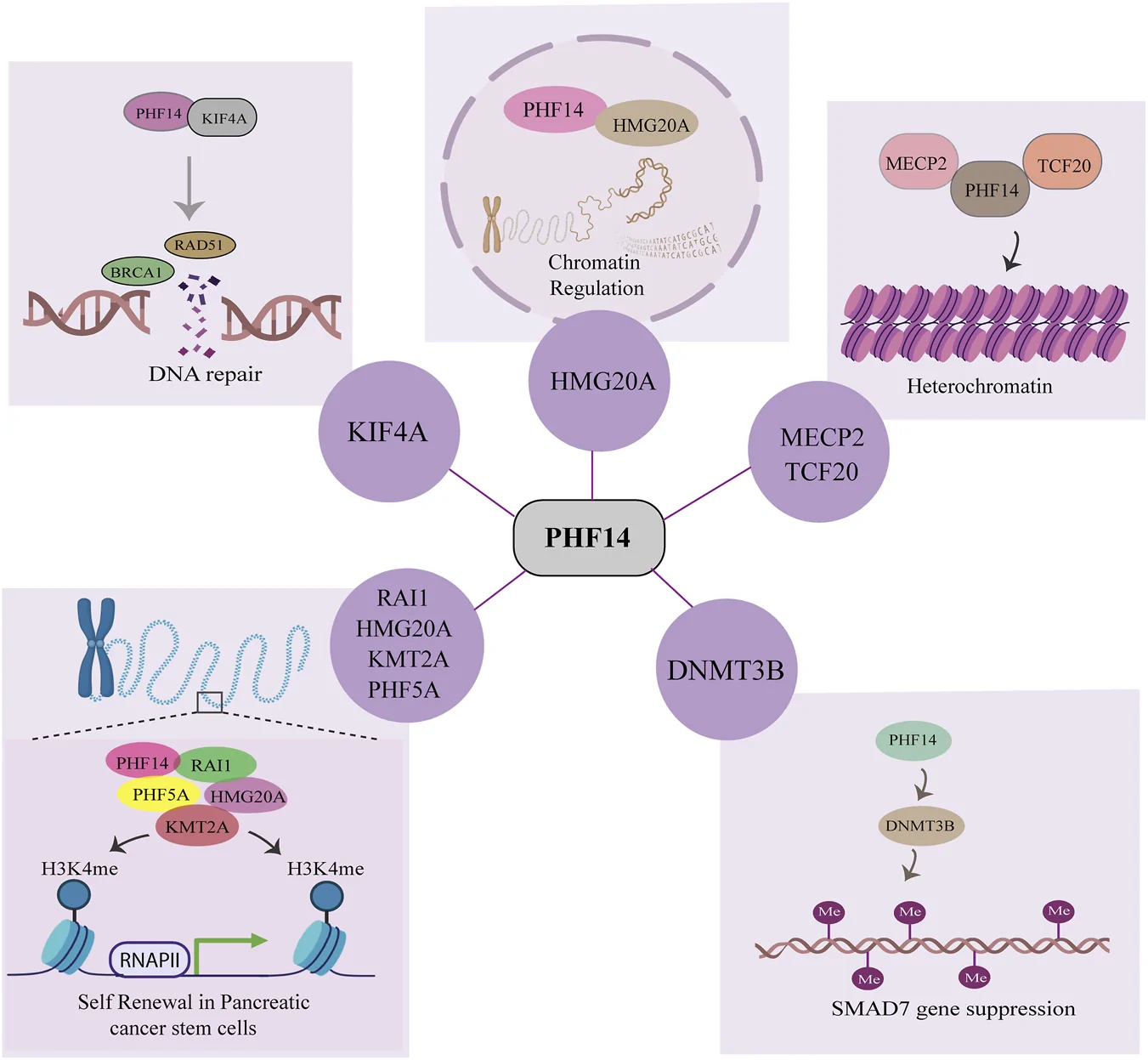
Summarization of PHF14 Interaction with other proteins and their biological role.

PHF14 interacts with various chromatin-associated proteins, which are associated with neurodevelopment processes and form distinct complexes, namely, MECP2-PHF14-TCF20, PHF14-HMG20A, KMT2A-PHF5A-PHF14-HMG20A-RAI1 (Dominguez et al., 2024) (Figure 2).

#### 2.2.1 MECP2-PHF14-TCF20 complex

The MeCP2-PHF14-TCF20 complex showed a critical role in brain function. Mutations of Methyl-CpG-binding protein 2 (MECP2) and Transcription factor 20 (TCF20) are associated with various neurodevelopmental disorders (Gonzales and LaSalle, 2010; Torti et al., 2019). It was shown that mutations affecting MeCP2-PHF14-TCF20 interaction resulted in neurological defects, including intellectual disability, speech delay or loss, autistic behaviours, motor incoordination, sleep disturbance, and regression (Zhou et al., 2022). The Methyl-CpG Binding Domain (MBD) of MECP2, which binds methylated DNA, mediates the interaction with TCF20. TCF20, in turn, interacts with PHF14 through its C-terminal histone-binding PHD domain. Within PHF14, the PHD1–2 domains bind to MECP2 and TCF20, wherein PHF14 plays a main role in connecting MECP2 and TCF20. Through this complex, MECP2 carries TCF20 to methylated heterochromatin. The coexpression of TCF20 and MECP2 was observed in MECP2-mediated synapse formation (Zhou et al., 2022). Both TCF20 and MECP2 have common downstream pathways and genes in neurons. Further, behavioral defects resulting from high MECP2 expression were overcome by decreasing TCF20 expression.

#### 2.2.2 PHF14-HMG20A

HMG20A is involved in neuronal differentiation (Wynder et al., 2005). The coiled coil regions of PHF14 and HMG20A interact in a way to form a leucine zipper motif, forming a stable complex in the nucleus (Gómez-Marín et al., 2022). In cells with PHF14 depletion, localization and levels of HMG20A are affected. It was demonstrated that PHF14, together with HMG20A, may also prefer ground or poised chromatin regions, as it was enriched with Histone H2A.Z (Gómez-Marín et al., 2022). Both proteins share a subset of transcriptional targets, reflecting coordinated chromatin-associated functions.

#### 2.2.3 KMT2A-PHF5A-PHF14-HMG20A-RAI1 complex

Followed by the PHF14-HMG20A interaction, it was identified that KMT2A-PHF5A-PHF14-HMG20A-RAI1 forms a complex. PHD finger-like domain-containing protein 5A (PHF5A) is identified as a nuclear interactor of PHF14 in pancreatic cancer stem cells (PCSC) and is a crucial component of the Splicing Factor 3b (SF3B) splicing complex, emerging as a new oncotarget (Li X. et al., 2023). Both PHF5A and PHF14 are chromatin-associated PHD-finger proteins known for reading histone modifications. Proteome and network analysis revealed that PHF5A and PHF14 function coordinately with HMG20A–RAI1. Transcription factor, Retinoic acid-induced protein 1 (RAI1), regulates circadian rhythm and its mutation is associated with Smith-Magenis syndrome (Falco et al., 2017; Chang et al., 2024). PHF5A-PHF14-HMG20A-RAI1 protein subcomplex can interact with histone through their different domains, but cannot modulate gene expression. They require epigenetic regulatory enzymes, such as Histone-lysine N-methyltransferase 2A (KMT2A), which interacts with the PHF5A-PHF14-HMG20A-RAI1 complex. KMT2A makes H3K4me3 that maintains self-renewal and tumorigenicity of PCSC (Mouti et al., 2023).

### 2.3 Regulation of PHF14

The PHF14 expression is regulated at multiple levels to control its function under specific biological conditions. SP4 is one of the transcription factors that acts as an upstream activator of PHF14 by directly binding to its promoter, and elevated expression of both SP4 and PHF14 in esophageal squamous cell carcinoma (ESCC) promotes cell proliferation while suppressing apoptosis (Wei et al., 2024). Similarly, phosphorylated SMAD3, a key mediator of TGF-β signaling, also functions as a transcription factor by binding the proximal upstream region of the PHF14 transcription start site (Yang et al., 2017).

Further, at the post-transcriptional level, PHF14 expression was shown to be regulated by non-coding RNAs. In bladder cancer, PHF14 mRNA was identified as a target of miR-590, which directly binds to it and suppresses its expression. But LINC00612, a long non-coding RNA, sponges miR-590 and decreases its availability to target PHF14 mRNA. Therefore, LINC00612 plays a role in upregulating PHF14 expression, and the LINC00612/miR-590/PHF14 axis was shown to enhance proliferation and epithelial-mesenchymal transition (EMT) in bladder cancer (Miao et al., 2019).

Post-translational modifications serve as a crucial mechanism for protein regulation. At the post-translational level, it is reported that the phosphorylation of PHF14 at the T287/S290 and S302 residues is significantly increased in colorectal cancer tissues compared to normal tissues (Pan et al., 2022). However, the other post-translational modifications of PHF14, namely, ubiquitination, SUMOylation, and various other PTMs, remain largely unexplored and warrant further investigation.

## 3 Molecular functions of PHF14

### 3.1 Role of PHF14-PDGFRα regulation in mesenchymal proliferation

PHF14 plays a role in the regulation of cell surface receptor tyrosine kinase Platelet-derived growth factor receptor alpha (PDGFRα) expression. PDGFRα is emerging as a mesenchymal stem cell marker that contributes to the progression of lung fibrosis (Farahani and Xaymardan, 2015; Biasin et al., 2020). Phf14 bound to the upstream of the PDGFRα gene and directly repressed its transcription in mesenchymal cells, renal fibroblast, embryonic fibroblast cells of mice, as well as in neuroblastoma cells (Kitagawa et al., 2012; Zhang et al., 2022). When PHF14-depleted cells were treated with Sunitinib, a PDGFR inhibitor, they exhibited increased sensitivity to the drug, showing a marked reduction in cell proliferation compared to control cells (Zhang et al., 2022). Regulation of PDGFRα is essential for lung mesenchymal cell proliferation during development. Even though Phf14 has no conserved domains like other transcriptional repressors, it acts as a transcriptional repressor of PDGFRα. The PZP domain of PHF14 preferentially binds to the unmodified histones and maintains the PDGFRα promoter in the repressed state. The coiled coil domain of PHF14 interacts with transcription factors that contain leucine zipper domains and is involved in the trans-repression of PDGFRα in mesenchymal cells. Gómez-Marín et al. demonstrated that the chromatin-associated protein, HMG20A, forms a complex with PHF14 and acts as a transcriptional repressor for many target genes including PDGFRα (Gómez-Marín et al., 2022; Kitagawa et al., 2012). Regulation of PDGFRα is also essential for lung mesenchymal cell proliferation during development.

### 3.2 PHF14 in cell cycle

PHF14 has been implicated in cell cycle regulation across multiple studies. During stress conditions like hypoxia, the cell cycle is adapted to promote survival factors (Ortmann et al., 2014). PHF14 expression was reduced under hypoxic conditions in cancer cells. Its inhibition led to upregulation of certain cell cycle inhibitors, causing cell cycle arrest at the G1-to-S phase transition, showing its involvement in the early stress response through epigenetic regulation (Park et al., 2019).

Similarly, in gastric cancer cells, PHF14 downregulation inhibited the expression of cell cycle-related proteins, cyclin D1 and Cyclin-dependent kinase 6 (CDK6), and suppressed colony formation *in vitro* and tumorigenesis *in vivo* (Zhao et al., 2020). CDK6 and cyclin D1 are crucial proteins required for G1 to S phase progression (Tadesse et al., 2015). PHF14 silencing across several lung cancer cell lines prolonged M phase, caused cell mitotic defects, and inhibited cell proliferation. Knockdown of PHF14 suppressed carcinogenesis and cancer cell growth, while overexpressing PHF14 promoted cell proliferation (Zhang et al., 2017).

In addition, PHF14 suppressed the Cyclin-Dependent Kinase Inhibitor 1A (CDKN1A) expression by controlling the level of H3K4me3 (Zhang L. et al., 2020). p21(CDKN1A) primarily hinders cell cycle progression at G1/S transition, inhibiting CDK2/cyclin-E and CDK4,6/cyclin-D (Karimian et al., 2016). In germinal centres, when PHF14 was depleted, there was an increase in H3K4me3 level, resulting in high p21 expression and reduced B-cell proliferation. PHF14 exhibited an effective role in B-cell proliferation (Zhang L. et al., 2020). Therefore, PHF14 is involved in cell cycle regulation through controlling epigenetic expression of cell cycle-related proteins.

### 3.3 Role of PHF14 in DNA damage response

DNA damage response (DDR) is identified as a novel function of PHF14 through its interaction with Kinesin family member 4A (KIF4A), which forms a functional complex in lung cancer and colorectal cancer (Zhang et al., 2017; Pan et al., 2022). KIF4A is a chromosome-associated kinesin motor protein that moves along microtubules using ATP. It regulates chromosome segregation and condensation in cell division (Wu and Chen, 2008; Vukušić et al., 2021). Both are localized in the nucleus in interphase and on chromatin during mitosis. Their interaction was mediated by 1–160 amino acids at the N-terminal region of PHF14 and the stalk domain of KIF4A (Zhang et al., 2017). Loss of PHF14 and KIF4A resulted in mitotic defects. It was observed that the PHF14–KIF4A interaction was essential for the formation as well as the recruitment of Breast cancer type 2 susceptibility protein (BRCA2) and DNA repair protein RAD51 homolog 1 (Rad51) foci. Assembly of these proteins at DNA damage sites is a critical process in homologous recombination-mediated DNA repair (Davies et al., 2001). Especially in colorectal cancer, PHF14 depletion decreased KIF4A expression, but BRCA2 levels were only minimally affected (Pan et al., 2022). Cell apoptosis increased following the knockdown of PHF14 in colorectal cancer cells (CRC) cells, along with a significant rise in γ-H2A.X levels, which confirmed the buildup of DNA damage. Elevation in the levels of γ-H2A.X, Serine/threonine-protein kinase Chk1 (CHK1), and phosphorylated (Serine/threonine-protein kinase ATR) ATR showed the activation of the ATR–CHK1–H2A.X-axis (Pan et al., 2022). Together, these findings demonstrated that PHF14 plays a role in the DNA damage response by mediating the recruitment of DNA repair proteins.

In pan cancer analysis, PHF14 expression showed a significant association with five common mismatch repair pathway genes (Epithelial cell adhesion molecule (EPCAM), Mismatch repair endonuclease PMS2 (PMS2), DNA mismatch repair protein Msh6 (MSH6), DNA mismatch repair protein Msh2 (MSH2), and DNA mismatch repair protein Mlh1 (MLH1)) in multiple The Cancer Genome Atlas tumors (TCGA) (Cao et al., 2023). PHF14 may maintain the viability of tumor cells by up-regulating genes in the DNA mismatch repair pathway (Cao et al., 2023). Furthermore, DNA repair-associated DEAD-Box Helicase 5 (DDX5) protein induced circular RNA of PHF14 in gastric cancer (Wang J. et al., 2023). DDX5 promotes cancer progression by activating DNA repair pathways (Le et al., 2023). circPHF14 enhanced tumorigenesis and growth in DDX5-positive gastric cancer cells.

### 3.4 Epigenetic regulation by PHF14

PHF14 plays a role in epigenetic regulation in association with DNA (cytosine-5)-methyltransferase 3B (DNMT3B), a *de novo* DNA methyltransferase responsible for cytosine methylation at CpG dinucleotides during early embryonic development (Zhang H. et al., 2020). An α-helix region of the ePHD domain (429–435aa) in PHF14 specifically binds with a CG-rich DNA motif, which enables PHF14 to read CG-rich elements in DNA. It recruits DNMT3B through its middle domain to hypermethylate the Smad7 gene. PHF14 connects DNMT3B and CG-rich regions, enhancing DNMT3B-mediated methylation on the Mothers against decapentaplegic homolog 7 (SMAD7) locus (Tian et al., 2023).

## 4 Key signaling pathways associated with PHF14

### 4.1 Wnt pathway

**FIGURE 3:**
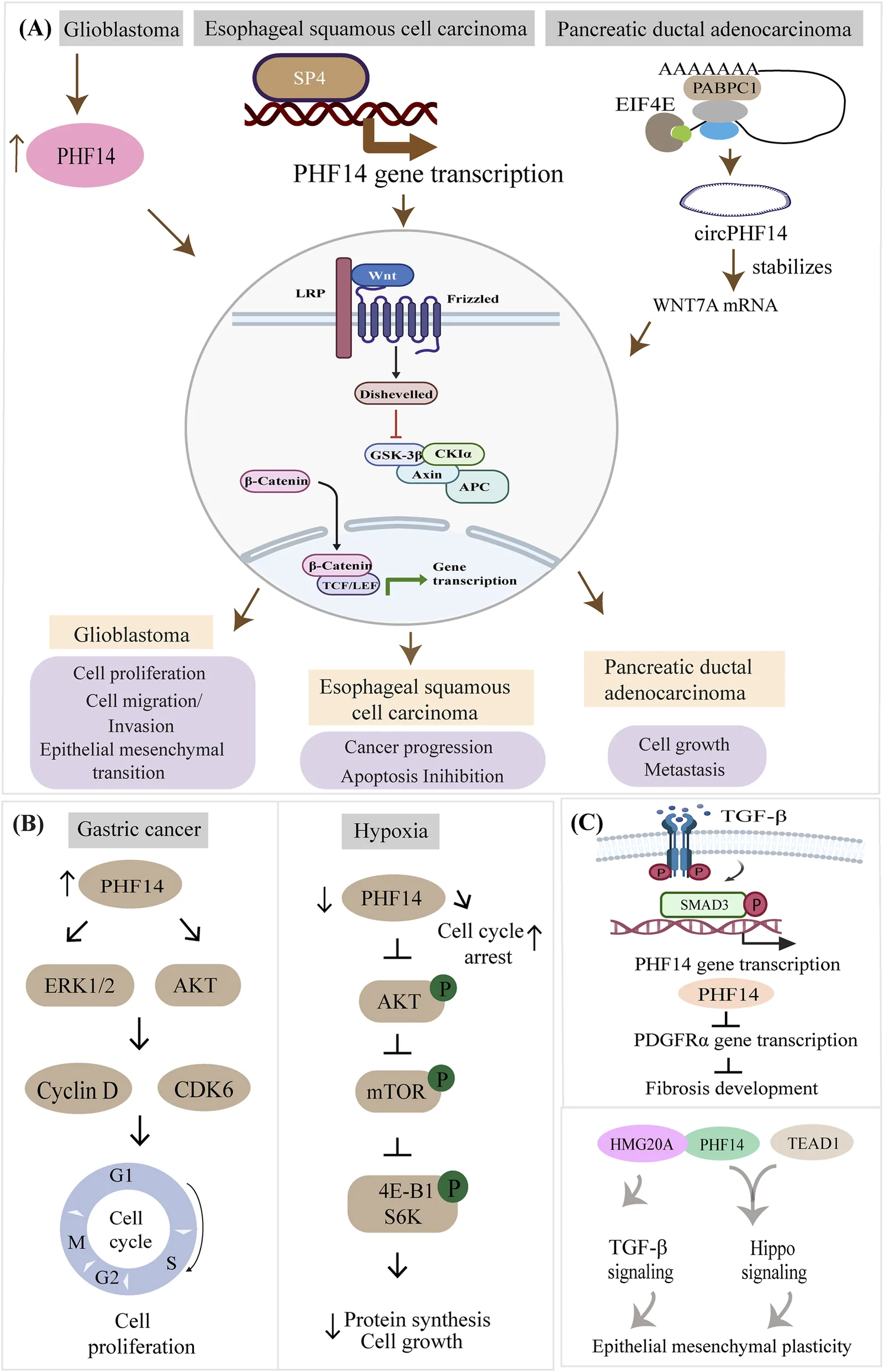
Role of PHF14 in different signaling pathways. **(A)** Wnt signaling, **(B)** Akt and ERK1/2 pathway, and **(C)** TGF-β and Hippo pathway.

PHF14 was reported to influence the Wnt/β-catenin signaling pathway, particularly in cancers such as glioblastoma, ESCC, and Pancreatic ductal adenocarcinoma (PDAC) (Wu et al., 2019; Wei et al., 2024; Fang et al., 2025) (Figure 3A). From these studies, it is evident that PHF14 is known to be upstream regulation of the Wnt signaling pathway. Wnt signaling contributes to carcinogenesis through controlling proliferation and metastasis (Zhan et al., 2017). In glioblastoma, PHF14-dependent regulation of the Wnt signaling pathway promoted cell proliferation, migration, and invasion of cancer cells (Wu et al., 2019). Increased transcription of PHF14 through Specificity protein 4 (SP4) stimulated Wnt signaling in ESCC (Wei et al., 2024). Wnt family member 7A (WNT7A), one of the ligands that can activate the canonical Wnt pathway, is stabilized at the mRNA level by circPHF14 in PDAC (Fang et al., 2025). CircPHF14 interacts with polyadenylate-binding protein cytoplasmic 1 (PABPC1), which binds to the 3′ poly A tail of mRNA to regulate its stability (Qi et al., 2022).

### 4.2 AKT and ERK1/2 pathway

PHF14 was identified to activate the AKT pathway and the ERK1/2 pathways in gastric cancer (Figure 3B). PHF14 silencing reduces the levels of phosphorylated ERK1/2 and AKT (Zhao et al., 2020). Both AKT and ERK pathways significantly contribute to gastric cancer through promoting proliferation, metastasis, and resistance (Wu et al., 2014; Lei et al., 2022). It has been demonstrated that the PHF14 induced AKT and ERK1/2 pathways promoted gastric cancer cell proliferation (Zhao et al., 2020). Under hypoxic stress, AKT–mTOR–4E-BP1/pS6K signaling was decreased following PHF14 inhibition. The loss of PHF14 led to low phosphorylation levels of key proteins involved in protein translation, including AKT (S473), Eukaryotic translation initiation factor 4E-binding protein 1 (4E-BP1) (T37/46), Serine/threonine-protein kinase mTOR (mTOR) (S2448), and Ribosomal protein S6 kinase beta-1 (p70S6K) (T389) (Park et al., 2019). This subsequently resulted in reduced protein synthesis and cell growth since AKT–mTOR–4E-BP1/pS6K signaling is essential for protein translation. Thus, PHF14 promotes both pathways, but the resultant biological process may vary depending on the context.

### 4.3 TGF-β and hippo pathway

TGF-β pathway is a regulator of EMT, which is a crucial developmental process in morphogenesis and organogenesis (Wang X. et al., 2023). Transforming Growth Factor Beta (TGF-β) signaling activated the transcription factor, Mothers against decapentaplegic homolog 3 (SMAD3), which enhanced Phf14 transcription in renal fibrosis (Yang et al., 2017). In contrast, Phf14 prevents fibrotic progression by suppressing PDGFRα (Kitagawa et al., 2012).

Furthermore, it affected the epithelial-mesenchymal transition induced by TGF-β signalling in lung adenocarcinoma (Tian et al., 2023). The absence of PHF14 increased epithelial markers, including E-cadherin and Tumor protein 63 (TP63). In complex with HMG20A, PHF14 interacts with Transcriptional enhancer factor TEF-1 (TEAD1), which is the main downstream effector of Hippo signaling (Gómez-Marín et al., 2022). The target genes of YAP/TAZ-TEAD including, Integrin beta-2 (ITGB2), Baculoviral IAP repeat-containing protein 5 (BIRC5), Insulin-like growth factor-binding protein 3 (IGFBP3), and Fibroblast growth factor 1 (FGF1) were reduced in HMG20A KD or PHF14 KD cells (Gómez-Marín et al., 2022). Transcription factor, TEAD1, promotes EMT in different cancers (Shen et al., 2022; Deng et al., 2024). Therefore, PHF14/HMG20A complex facilitates pathways connected to epithelial-mesenchymal plasticity (Figure 3C).

## 5 Pathological implications of PHF14

### 5.1 Genetic variants of PHF14 and its disease associations

**FIGURE 4:**
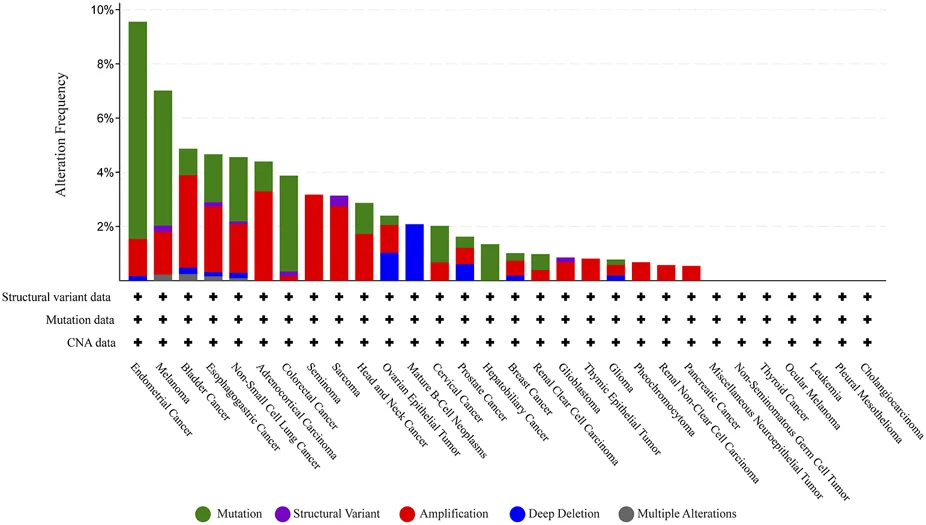
Genetic variations of PHF14 across cancer types from cBioPortal (Cerami et al., 2012). Stacked bars show the frequency and types of PHF14 variations, including mutations (green), structural variants (purple), amplifications (red), deep deletions (blue), and multiple alterations (grey) across the TCGA Pan-Cancer study.

Distinct genetic variants of PHF14, such as copy number variations and single-nucleotide polymorphisms (SNPs), have been reported in various biological contexts (Table 1). Previous studies suggested that the candidate genes for spinocerebellar ataxia 21 are located within the linked chromosomal interval 7p21–p15, where the PHF14 locus is present (Vuillaume et al., 2002). Later, mutations in the Transmembrane protein 240 (TMEM240) gene were identified to be associated with spinocerebellar ataxia 21 (Homa et al., 2020; Cherian et al., 2022). A single nucleotide polymorphism, PHF14 K115R (rs218966), was identified as one of the candidate variants linked to stroke risk in sickle cell anemia patients using whole exome sequencing (Flanagan et al., 2013). But this variant failed to show statistical significance in a follow-up validation study. Another study done by Friedrichs et al. showed that the rs218966 marker was associated with hypotension based on a large family study of Mexican Americans (GAW19 data set) (Friedrichs et al., 2016). It was reported that PHF14 mutations account for 18% of colorectal cancer patients (Pan et al., 2022). Additionally, genetic variation patterns across multiple cancers, based on data from cBioPortal, are shown in Figure 4 (Cerami et al., 2012). The frequency and the distribution of PHF14 genomic alteration categories (mutations, amplifications, deep deletions, structural variants, and multiple alterations) for each cancer type are provided in Supplementary Table S1. This pancancer study did not apply any statistical threshold. In the study conducted in Gallus gallus, SNP (AX-76063628), located in the PHF14 gene on chromosome GGA2, was often identified in sperm membranes that were damaged due to cryopreservation. The T allele at this locus was shown to adversely impact membrane integrity (Dementieva et al., 2025)

**TABLE 1:** Genetic variants of PHF14 reported in different pathological conditions.

Type of genetic variant	Findings	Context	References
Bi-allelic inactivating mutations	Mutations in these genes showed microsatellite instability	Human Colon cancer cell lines	Ivanov et al. (2007)
Somatic mutations; 3/21 (14%) tumors; Out of them, two were missense mutations and 4 caused splicing site losses	Loss of PHF14 function resulted in increased cell proliferation in several cell lines and in a neurocytoma primary culture	Human Neurocytomas using WES (whole exome sequencing)	Zhang et al. (2022)
Single-nucleotide polymorphism (rs218966)	Associated with hypotension	Human cohort (GAW19 data set)	Flanagan et al. (2013)
Copy number alterations	Increased expression; segmental amplifications and deletions on Chromosomes 7 and 12	Human ossifying fibroma (whole-genome shallow sequencing)	Ma et al. (2021)
Nonsense variant (c.1573A > T, p.R525*); missense variant (c.964T > G, p.C322G)	Inhibit PHF14 interactions with MeCP2 and TCF20	Human RTT-like neurological features	Zhou et al. (2022)
14.7 Mb interstitial deletion on chromosome 7	No significant association	Human neonate with Persistent pulmonary hypertension	Schinagl et al. (2017)
Homozygous deletion at chromosomal region 7p21.3	No detection of PHF14mRNA and PHF14 protein	Human Biliary tract cancer cells	Akazawa et al. (2013)
Deletions and duplication on chromosome 7p21.3	Dandy-Walker malformations	Human Dandy-Walker syndrome	Liao et al. (2012a)
Phf14 knockout (mouse model)	Inhibit renal fibrotic progression; Suppress PDGFRα expression	Mouse models of lung and renal fibrosis	Kitagawa et al. (2012), Yang et al. (2017)

The *PHF14* gene is located at 7p21.3, which is linked with Dandy-Walker syndrome (Liao et al., 2012a; 2012b). Using Array-based comparative genomic hybridization (array-CGH), microdeletions/duplications identified in fetal patients with Dandy–Walker malformation were mapped to the chromosomal locus 7p21.3, which includes PHF14 and Cytochrome c oxidase subunit NDUFA4 (NDUFA4) (Liao et al., 2012b). Dandy-Walker syndrome is a rare congenital malformation characterized by enlargement of the posterior fossa, dilatation of the fourth ventricle, and cerebellar hypoplasia with upward rotation (Lomi et al., 2025). More information is necessary to fully understand how the detected genetic variations of PHF14 contribute to the pathophysiology of the disorders.

### 5.2 Role of PHF14 in cancer

**FIGURE 5:**
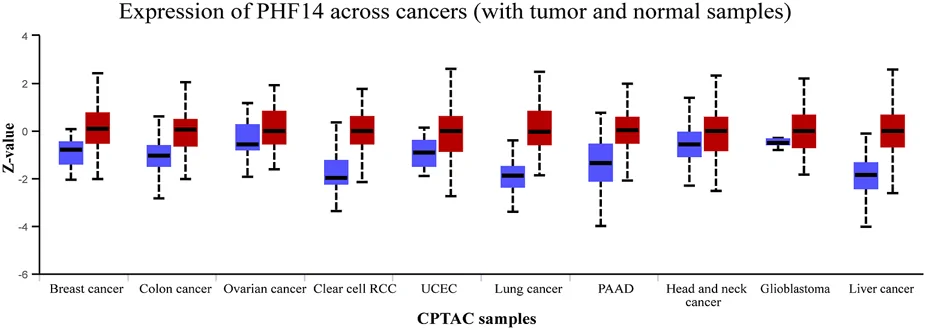
PHF14 protein expression across cancers based on CPTAC (Clinical Proteomic Tumor Analysis Consortium) proteomics data from the UALCAN (The University of ALabama at Birmingham CANcer data analysis Portal) database (Chandrashekar et al., 2022). Boxplots show PHF14 expression (Z-values) in tumor (red) and normal (blue) tissues across multiple cancer types, indicating higher PHF14 levels in most tumors.

PHF14 has been implicated in the development of several cancers (Table 2). PHF14 was highly expressed in Undifferentiated spindle cell sarcoma (UCSC), lung cancer, and gastric cancer (Zhang et al., 2017; Zhao et al., 2020; Wei et al., 2025), and its expression across different cancers based on CPTAC (Clinical Proteomic Tumor Analysis Consortium) proteomics data from the UALCAN (The University of ALabama at Birmingham CANcer data analysis Portal) database is shown in Figure 5 (Chandrashekar et al., 2022). Systematic analysis of PHF14 expression revealed that it positively correlated with three DNA methyltransferases, which highlights the epigenetic role of PHF14 in cancer (Mouti et al., 2023). But the molecular mechanism behind the role of PHF14 varies with different cancers. It is linked with cell cycle-regulated proteins in gastric cancer (Zhao et al., 2020), epigenetic regulation of genes like SMAD7 (Tian et al., 2023) and circular PHF14 (non-coding RNA) in gastric cancer tumorigenesis (Wang J. et al., 2023), and PDAC (Pan et al., 2022; Wei et al., 2024; Fang et al., 2025). Pan-cancer analysis of PHF14 indicated that its expression was correlated with the prognosis of certain cancers (Cao et al., 2023). In particular, PHF14 was suggested as a potential biomarker in lung cancer (Zhang et al., 2017), nasopharyngeal carcinoma (Chang et al., 2025), and a therapeutic target in PDAC, ESCC, and colorectal cancer (Pan et al., 2022; Wei et al., 2024; Fang et al., 2025). Like other PHD finger proteins, it contributes to oncogenesis through regulation of chromatin structure and different signaling pathways (Fan et al., 2024). Thus, along with bromodomain-directed therapeutic interventions for cancer, drugs targeting PHF14 may also serve as a potential therapeutic target (Jothi et al., 2026). Although the druggability of PHF14 remains largely unexplored, potential therapeutic strategies may include disrupting PHF14-mediated interactions, altering its expression using RNA-based approaches, or targeting upstream regulatory mechanisms. But, the development of epigenetic therapeutics requires highly selective targeting to minimize off-target effects and preserve normal cellular functions (Dai et al., 2024). In addition, the molecular interactome of PHF14 remains incompletely characterized. Moreover, the molecular mechanisms underlying PHF14 function are not yet fully understood, as many of its interacting partners remain unidentified. The lack of complete understanding of the PHF14 interactome presents a significant challenge for the rational design of targeted therapies.

**TABLE 2:** Role of PHF14 in multiple cancers.

Type of cancer	Findings	References
Lung cancer	PHF14 was identified as one of the risk genes correlated with poor survival; High expression linked with poor survival; Bound and co-localized with KIF4A; enhanced cell proliferation	Zhang et al. (2017), Chen et al. (2021)
GBM (glioblastoma multiforme)	High expression of PHF14; PHF14 silencing showed inhibited migration, invasion, proliferation, and enhanced cell apoptosis	Wu et al. (2019)
Colorectal cancer	Increased PHF14 expression correlates with poor prognosis; Interact with KIF4A; Involved in formation of BRCA2/Rad51; mediated ATR-CHK1-H2A.X pathway activation; Promoted cell proliferation and growth	Pan et al. (2022)
Lung adenocarcinoma	Bound DNMT3B and mediated transcriptional suppression of SMAD7 by DNA methylation, resulting in TGF-β pathway activation; promoted metastasis	Tian et al. (2023)
Bladder cancer	LINC00612 sponge miR-590 which prevents its suppression on PHF14 transcription; High PHF14 expression enhanced epithelial mesenchymal transition (EMT)	Miao et al. (2019)
Undifferentiated spindle cell sarcoma (UCSC)	PHF14 showed high expression in low-grade UCSCs	Wei et al. (2025)
Pancreatic ductal adenocarcinoma (PDAC)	circPHF14 upregulation; Stabilized WNT7A mRNA through PABPC1 interaction; Activation of Wnt/β-catenin pathway; Promoted cancer growth and metastasis	(Fang et al., 2025)
Esophageal Squamous Cell Carcinoma (ESCC)	PHF14 transcription activated by SP4; Enhanced cell proliferation and suppresses apoptosis	Wei et al. (2024)
Pancreatic cancer stem cells (PCSCs)	PHF5A-PHF14-HMG20A-RAI1 protein subcomplex interacted with KMT2A; Supported self-renewal capacity, cell viability, and *in vivo* tumorigenicity	Mouti et al. (2023)
Soft tissue sarcoma	PHF14 is identified as one of the zinc finger proteins associated with the prognosis of soft tissue sarcoma	Li et al. (2023a)
Pan-cancer analysis	PHF14 expression closely linked to carcinogenesis and prognosis of specific tumors; Potential involvement in tumor immunity by modulating immune checkpoint gene expression	Cao et al. (2023)
Gastric cancer	circPHF14 necessary for tumorigenesis in DDX5-positive gastric cancer cells; High PHF14 expression affected patient survival; Increased cell proliferation; Decreased PHF14 expression reduced cell cycle proteins and phosphorylated AKT and ERK1/2 levels	Zhao et al. (2020), Wang et al. (2023a)
Biliary tract cancer	PHF14 knockdown increased cell growth	Akazawa et al. (2013)
Neurocytoma	Loss of PHF14 promoted proliferation and colony formation; Repressed PDGFRα expression and increased sensitivity to treatment with the PDGFR inhibitor Sunitinib	Zhang et al. (2022)
Ossifying sarcoma	Amplified PHF14 expression due to copy number alteration	Ma et al. (2021)
Nasopharyngeal carcinoma	Reduced PHF14 caused decreased cell migration, proliferation, and invasion; Correlated with immune infiltration	Chang et al. (2025)

### 5.3 Contribution to fibrosis, developmental defects, and immune regulation

The role of PHF14 in fibrotic disease is context-dependent. Studies in lung and kidney fibrosis reported that PHF14 showed a protective effect by limiting excessive mesenchymal cell proliferation and fibrotic progression (Kitagawa et al., 2012; Yang et al., 2020). In mouse models of interstitial lung development and fibrosis, Phf14 regulates mesenchyme growth by regulating PDGFRα expression. Absence of Phf14 in mesenchymal fibroblasts showed high PDGFRα expression and proliferation (Kitagawa et al., 2012). This is because Phf14 directly inhibits PDGFRα expression as a transcription factor. Conversely, in lung adenocarcinoma (LAD) PHF14 binds to DNMT3B and acts as a DNA CpG motif reader, recruiting DNMT3B to the SMAD7 gene locus, resulting in DNA methylation and transcriptional repression of SMAD7. SMAD7 causes hyperactivation of TGF-β signaling and promotes LAD metastasis rather than preventing fibrosis (Tian et al., 2023).

In the case of renal fibrosis, Phf14-deficient mice had more severe effects. In acute kidney fibrosis mouse model induced by folic acid, Phf14 was persistently upregulated during renal fibrosis through TGF-β/SMAD3 signaling. Upon TGF-β stimulation, Smad3 gets phosphorylated, and the phosphorylated Smad3 enhances Phf14 expression by binding to the transcription start site of Phf14. The upregulated Phf14 limits PDGFRα-mediated proliferation of fibroblasts, thereby providing a protective response (Yang et al., 2017). As fibrosis progresses to the chronic stage, epigenetic alterations play a major role in sustaining fibroblast activation. TGF-β upregulates DNA methyltransferase 3A (DNMT3A) and DNA methyltransferase 1 (DNMT1) through SMAD-dependent signaling, resulting in promoter hypermethylation and silencing of the suppressor of cytokine signaling 3 (SOCS3). The suppression of SOCS3 mitigates STAT3 inhibition, leading to the hyperactivation of STAT3 and fibrotic progression (Dees et al., 2020).

Neonatal mice with a knockout of the Phf14 gene died shortly after birth, as a result of respiratory failure. This study reinforced its significant role in the multiorgan development in mice (Huang et al., 2013). In addition, its role in humoral immunity is evident as it promotes the B-cell proliferation germinal centre through p21 epigenetic regulation (Zhang L. et al., 2020).

## 6 Conclusion and future perspectives

PHF14 is a multi-PHD finger histone-binding protein that functions as an epigenetic regulator. This review provided a comprehensive overview of the molecular interaction and functional role of PHF14. PZP-mediated bipartite recognition, as discussed in this review, explains its interaction with histones and its preferential binding properties. The structural properties of PHF14 and its interaction with chromatin-associated proteins provide insights into its epigenetic regulatory role, which may contribute to various key cellular processes and disease-specific outcomes. This review also highlights the role of PHF14 in several cancers and its significant contribution to oncogenesis. Collectively, these findings position the PHF14 protein as a key molecular regulator integrating signaling pathways, epigenetic regulation, and disease pathogenesis, warranting the need for further mechanistic and translational investigation.

Despite these findings, future research should investigate the molecular mechanisms by which PHF14 promotes cancer growth and influences cancer biology. Furthermore, the regulatory mechanisms of PHF14, including post-translational modifications and subcellular localization, remain underexplored and may influence its activity. The identification of its full-length structure would provide more insights into how the PHD zinc fingers interact within these complexes and read histone modifications. Given its involvement in cancer and its interactors in neurodevelopmental disorders, elucidating its molecular features may reveal new therapeutic prospects. Overall, PHF14 appears to function as an important regulatory node linking molecular interactions, cellular homeostasis, signaling pathways, and disease progression.
