# Supplementary material for: Current insights into PHF14: molecular features and functional roles
Source: Front Mol Biosci. 2026 Aug 21;13:1858432. doi: 10.3389/fmolb.2026.1858432 (PMC13541444; doi:10.3389/fmolb.2026.1858432)
Supplement: Supplementary file 1 [file Table1.docx]

Title: Current insights into PHF14: Molecular Features and Functional Roles

**Supplementary Table S1**. Frequency and distribution of PHF14 genomic alterations across TCGA PanCancer Atlas datasets retrieved from cBioPortal.

| **Cancer types** | **Patients (n)** | **Overall PHF14 alteration (%)** | **Mutation (%) [n]** | **Amplification (%) [n]** | **Deep deletion (%) [n]** | **Structural variant (%) [n]** | **Multiple alterations (%) [n]** |
| --- | --- | --- | --- | --- | --- | --- | --- |
| Uterine Corpus Endometrial Carcinoma | 529 | 10.40 | 8.88 (47) | 1.32 (7) | 0.19 (1) | – | – |
| Skin Cutaneous Melanoma | 442 | 7.01 | 4.98 (22) | 1.58 (7) | – | 0.23 (1) | 0.23 (1) |
| Lung Adenocarcinoma | 566 | 6.36 | 3.18 (18) | 2.65 (15) | 0.35 (2) | 0.18 (1) | – |
| Bladder Urothelial Carcinoma | 411 | 4.87 | 0.97 (4) | 3.41 (14) | 0.24 (1) | – | 0.24 (1) |
| Uterine Corpus Endometrial Carcinoma | 529 | 10.40 | 8.88 (47) | 1.32 (7) | 0.19 (1) | – | – |
| Skin Cutaneous Melanoma | 442 | 7.01 | 4.98 (22) | 1.58 (7) | – | 0.23 (1) | 0.23 (1) |
| Lung Adenocarcinoma | 566 | 6.36 | 3.18 (18) | 2.65 (15) | 0.35 (2) | 0.18 (1) | – |
| Bladder Urothelial Carcinoma | 411 | 4.87 | 0.97 (4) | 3.41 (14) | 0.24 (1) | – | 0.24 (1) |
| Stomach Adenocarcinoma | 440 | 4.77 | 1.82 (8) | 2.27 (10) | 0.23 (1) | 0.23 (1) | 0.23 (1) |
| Adrenocortical Carcinoma | 91 | 4.40 | 1.10 (1) | 3.30 (3) | – | – | – |
| Esophageal Adenocarcinoma | 182 | 4.40 | 1.65 (3) | 2.75 (5) | – | – | – |
| Colorectal Adenocarcinoma | 594 | 3.87 | 3.54 (21) | 0.17 (1) | – | 0.17 (1) | – |
| Sarcoma | 255 | 3.14 | – | 2.75 (7) | – | 0.39 (1) | – |
| Head and Neck Squamous Cell Carcinoma | 523 | 2.87 | 1.15 (6) | 1.72 (9) | – | – | – |
| Lung Squamous Cell Carcinoma | 487 | 2.46 | 1.44 (7) | 0.82 (4) | – | – | 0.21 (1) |
| Ovarian Serous Cystadenocarcinoma | 584 | 2.40 | 0.34 (2) | 1.03 (6) | 1.03 (6) | – | – |
| Diffuse Large B-Cell Lymphoma | 48 | 2.08 | – | – | 2.08 (1) | – | – |
| Cervical Squamous Cell Carcinoma | 297 | 2.02 | 1.35 (4) | 0.67 (2) | – | – | – |
| Uterine Carcinosarcoma | 57 | 1.75 | – | 1.75 (1) | – | – | – |
| Prostate Adenocarcinoma | 494 | 1.62 | 0.40 (2) | 0.61 (3) | 0.61 (3) | – | – |
| Liver Hepatocellular Carcinoma | 372 | 1.34 | 1.34 (5) | – | – | – | – |
| Testicular Germ Cell Tumors | 149 | 1.34 | – | 1.34 (2) | – | – | – |
| Breast Invasive Carcinoma | 1084 | 1.01 | 0.28 (3) | 0.55 (6) | 0.18 (2) | – | – |
| Kidney Renal Clear Cell Carcinoma | 511 | 0.98 | 0.59 (3) | 0.39 (2) | – | – | – |
| Glioblastoma Multiforme | 585 | 0.85 | – | 0.68 (4) | – | 0.17 (1) | – |
| Thymoma | 123 | 0.81 | – | 0.81 (1) | – | – | – |
| Brain Lower Grade Glioma | 514 | 0.78 | 0.19 (1) | 0.39 (2) | 0.19 (1) | – | – |
| Kidney Renal Papillary Cell Carcinoma | 283 | 0.71 | – | 0.71 (2) | – | – | – |
| Pheochromocytoma and Paraganglioma | 178 | 0.56 | – | 0.56 (1) | – | – | – |
| Pancreatic Adenocarcinoma | 184 | 0.54 | – | 0.54 (1) | – | – | – |
